# Covalent Labeling Automated Data Analysis Platform for High Throughput in R (coADAPTr): A Proteome-Wide Data Analysis Platform for Covalent Labeling Experiments

**DOI:** 10.1021/jasms.4c00196

**Published:** 2024-10-02

**Authors:** Raquel L. Shortt, Lindsay K. Pino, Emily E. Chea, Carolina Rojas Ramirez, Daniel A. Polasky, Alexey I. Nesvizhskii, Lisa M. Jones

**Affiliations:** † Department of Pharmaceutical Sciences, University of Maryland, Baltimore, Maryland 21201, United States; ‡ Talus Bio, Seattle, Washington 98122, United States; § GenNext Technologies, Half Moon Bay, California 94019, United States; ⊥ Department of Pathology, University of Michigan, Ann Arbor, Michigan 48109, United States; ¶ Department of Chemistry and Biochemistry, University of California San Diego, La Jolla, California 92093, United States

## Abstract

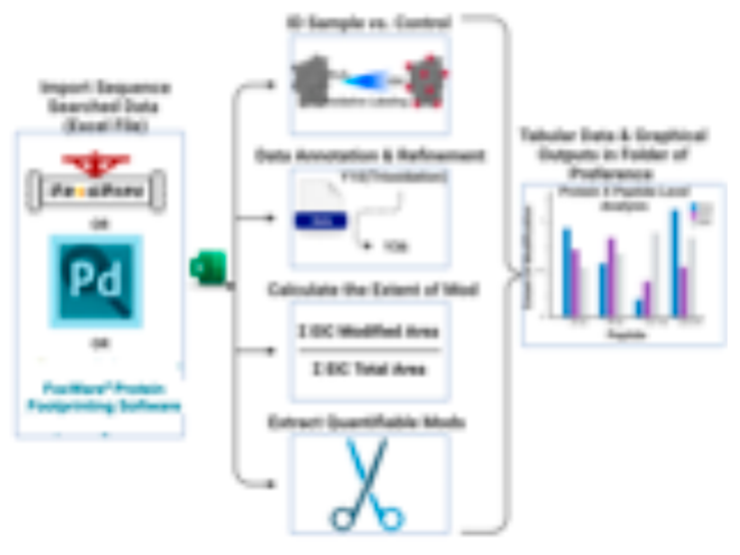

Covalent labeling methods coupled to mass spectrometry have emerged in recent years for studying the higher order structure of proteins. Quantifying the extent of modification of proteins in multiple states (i.e., ligand free vs ligand-bound) can provide information on protein interaction sites and regions of conformational change. Though there are several software platforms that are used to quantify the extent of modification, the process can still be time-consuming, particularly for proteome-wide studies. Here, we present an open-source software for quantitation called Covalent labeling Automated Data Analysis Platform for high Throughput in R (coADAPTr). coADAPTr tackles the need for more efficient data analysis in covalent labeling mass spectrometry for techniques such as hydroxyl radical protein footprinting (HRPF). Traditional methods like Excel’s Power Pivot (PP) are cumbersome and time-intensive, posing challenges for large-scale analyses. coADAPTr simplifies analysis by mimicking the functions used in the previous quantitation platform using PowerPivot in Microsoft Excel but with fewer steps, offering proteome-wide insights with enhanced graphical interpretations. Several features have been added to improve the fidelity and throughput compared to those of PowerPivot. These include filters to remove any duplicate data and the use of the arithmetic mean rather than the geometric mean for quantitation of the extent of modification. Validation studies confirm coADAPTr’s accuracy and efficiency while processing data up to 200 times faster than conventional methods. Its open-source design and user-friendly interface make it accessible for researchers exploring intricate biological phenomena via HRPF and other covalent labeling MS methods. coADAPTr marks a significant leap in structural proteomics, providing a versatile and efficient platform for data interpretation. Its potential to transform the field lies in its seamless handling of proteome-wide data analyses, empowering researchers with a robust tool for deciphering complex structural biology data.

## Introduction

Covalent labeling (CL) techniques coupled with mass spectrometry (MS) have allowed the interrogation of protein structures and interactions.^1,2^ In CL experiments, a protein’s surface is modified with a specific (i.e., glycine ethyl ester or diethylpyrocarbonate) or nonspecific label (i.e., deuterium or hydroxyl radical) to provide information on its higher order structure. These methods are coupled with liquid chromatography–mass spectrometry (LC-MS/MS) to identify and quantify labeling. Hydroxyl radical protein footprinting (HRPF), a nonspecific CL technique, has become a critical component in structural biology studies, and proven useful in delineating protein higher order structure, protein–protein interactions, and protein conformations.^3−10^ The analysis of covalent labeling mass spectrometry is dependent on both database sequence searching, to identify labeled amino acids, and quantitation of the extent of modification (EOM). HRPF is especially challenging for both database searching and EOM quantitation, owing to the high number of potential modification products on 19 out of 20 amino acids (Table S1). Further, fast photochemical oxidation of proteins (FPOP), an HRPF technique that uses laser photolysis of hydrogen peroxide (H_2_O_2_) to generate hydroxyl radicals, has recently been extended to the study of intact cells (IC-FPOP) and in an *in vivo* system (IV-FPOP).^11,12^ These methods modify hundreds to thousands of proteins in a single experiment for proteome-wide structural biology, further increasing the complexity of data analysis. A number of search algorithms have been successfully used for searching HRPF data including Sequest,^13^ Byonic,^14^ FoxWare Software, ProtMapMS,^15^ MSFragger,^16^ PEAKS,^17^ and Mascot.^18^

Calculating the EOM on the peptide- and residue-levels presents another challenge and can often be time-consuming and tedious. FPOP-based experiments are generally performed by comparing a protein in at least two different states. In addition, control samples where proteins are exposed to H_2_O_2_ but not laser irradiation are analyzed to observe the background oxidation. The EOM of these control samples is also calculated and then subtracted from the EOM of laser irradiated samples. This along with the multiple replicates required for appropriate statistical analysis increases the time for analyzing the FPOP data. FoxWare Software and ProtMapMS can directly perform peptide- and residue-level quantitation of HRPF data. The Protein Metrics suite of software can also be used for quantitation.^19^ However, all of these platforms require a license fee. Mass Studio is another platform that can be used to both search and quantify covalent labeling data.^20^ Though not used for HRPF, it has been successfully used for carbene footprinting another nonspecific covalent labeling technique. Nevertheless, none of these platforms have been applied to proteome-wide HRPF quantitation. Rinas et al. implemented a Power Pivot (PP) extension in Microsoft Excel to manually calculate modification extent at the peptide- and residue-level.^13^ Although it served as a solution to a complex problem, the workflow is time-consuming for proteome-wide experiments, highlighting major challenges for IC- and IV-FPOP.

To address these challenges, we developed an open-source tool using R programming titled Covalent labeling Automated Data analysis platform for high Throughput in R (coADAPTr). In R, we created a series of functions emulating existing Excel functions but with reduced mathematical steps. Sorting data by Master Protein Accession and adding parameters ensured analysis only of proteins detected in both differential conditions, thus enabling proteome-wide analysis. This package also integrates graphical functionalities for an enhanced interpretation. The vision for coADAPTr is to provide researchers with a convenient, open access means to analyze data from experiments investigating complex biological phenomena via HRPF and other covalent labeling MS methods.

## Experimental Section

### EOM Calculations

The fractional oxidation per peptide or residue was calculated according to the following eq (eq 1):
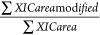
1For peptide-level analysis, extracted ion chromatogram (XIC) area modified is the area of the peptide with a modified residue(s), and XIC area is the total area of the same peptide with and without the modified residue(s). For residue-level analysis, the XIC area modified is the area of a modified residue and the XIC area is the total area of the modified residue and all unmodified residues in the peptide. The EOM is calculated from the subtraction of the EOM of a control sample (no laser irradiation) from the oxidized sample. The EOM calculation contains only a single charge state per peptide, as duplicate sequence entries are removed by coADAPTr.

### Sequence Searching Software

In vitro FPOP samples were searched by using FoxWare Software with Comet to identify the unmodified peptides. A precursor ion tolerance of 10 ppm and fragment tolerance of 1.0005 Da with a false discovery rate (FDR) of 1% was used. The monoisotopic mass of an identified unmodified peptide was used to calculate the monoisotopic masses for the modified peptides (+16 + 32, or +48 Da). XICs were generated for all of the peptides. The modified peptide chromatographic area was evaluated by using the MS1 spectra data at the XIC peak apex. The XIC peak was included in the EOM calculation if it eluted within the predicted retention time window around the corresponding unmodified peptide and if the isotopic distribution in the observed spectrum had a correlation score of ≥0.9 against the theoretical isotope patterns of averagine peptide models. Once the XIC peaks were evaluated and selected, FoxWare Software calculated the average peptide oxidation events (APO), which considers the number of modified amino acids per peptide, using the following eq (eq 2).
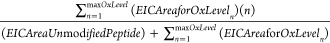
2

Spheroid-FPOP data was searched using Proteome Discoverer 2.5 (Thermo Fisher Scientific, Waltham, MA) with the Sequest algorithm as described previously.^21^ The files were searched against a SwissProt *Homo sapiens* database implementing a multilevel workflow to accommodate all possible FPOP modifications.^13^ The processing workflow contains five search levels, where FPOP modifications were dispersed across the individual search levels. The tolerance for fragment ions was 0.02 Da, while the parent ion tolerance was 10 ppm. Trypsin was set as the proteolytic enzyme used, and only one missed cleavage was considered. The false discovery rate (FDR) was set to 1% where proteins were only accepted if at least two distinct peptides were identified with the FDR filter. The resultant consensus files were exported as a Microsoft Excel file and analyzed by the PowerPivot add-in or coADAPTr. The XIC area was calculated from the precursor abundance reported in the consensus file.

IV-FPOP data was searched using FragPipe as described previously.^16^ Briefly, MSFragger searches were performed with FPOP-related modifications specified as variable modifications or mass offset. A maximum of 3 variable modifications were allowed per peptide, for a few FPOP modifications (oxidation at MFHILVWY), and a maximum of one for protein N-terminal acetylation. Carbamidomethylation of Cys was set as a fixed modification in all of the searches. All searches used MSFragger’s built-in mass calibration option, fully enzymatic cleavage with the strict-trypsin enzyme setting (max 2 missed cleavages), peptide lengths of 7–50 amino acids, and N-terminal Met clipping enabled. Other FPOP modifications were set as mass offsets. These searches used delta mass localization and reported mass offsets as variable modifications in the MSFragger output. An FDR of 1% was applied at PSM, peptide, and protein levels, using the group FDR method to calculate separate score thresholds for peptides with no modifications, common modifications (oxidation at M and N-terminal acetylation), and FPOP modifications.

### Programming Software

Functions and R package was built in RStudio “Chocolate Cosmos” Release (a00d0e77, 2024–04–24) for windows. The package was stored and managed using GitHub Bash downloaded on GitHub Desktop Version 3.3.14 (x64). The location of the package on GitHub is https://github.com/LJonesGroup/coADAPTr.git. Version control was set to “on” and managed by GitHub. The script used to adapt FragPipe PSM tables for coADAPTr analysis can be found at https://github.com/Nesvilab/FragPipe-to-coADAPTr. This capability will eventually be integrated into FragPipe and will be available without requiring an additional script after the next major release.

## Results and Discussion

### Evaluating the Existing Excel-Based Data Analysis Method

Though the previously established data analysis method in PP provided an advantage for calculating the extent of modification for proteome-wide HRPF experiments, the process is tedious and time-consuming and has the capacity to produce calculation inaccuracies. First, when the data are sorted/filtered by the sequence, there appears to be identical entries for some peptides with the only difference being the search node that mapped the peptide spectral match. This is a result of the multilevel sequence searching algorithm that was implemented in Proteome Discoverer (PD) to reduce computational search space while searching for the myriad of FPOP modifications. Since each node only searches for a subset of modifications, it is possible for unmodified species to be detected in each node, which would result in an under estimation of the total extent of modification since the denominator in eq 1 would be larger. Second, the extent of modification can still be calculated even if the peptide was detected in only one replicate. This reduces the fidelity of the data since statistical analysis of replicate samples cannot be performed. Finally, if the peptide was detected as modified, then PP would still present an extent of modification value, even if the unmodified peptide was not observed. This is due to PP forcing the presentation of a result based on the formulas implemented, even if all of the data necessary to quantify the extent of modification were not present. However, the control files, where samples are exposed to H_2_O_2_ but not laser irradiation, are essential to consider background oxidation and not obtain a higher than actual calculated EOM. For this reason, users generally parse through the data manually to ensure any reported modifications were indeed quantifiable thus increasing analysis time.

### Improvements in coADAPTr

**Figure 1:**
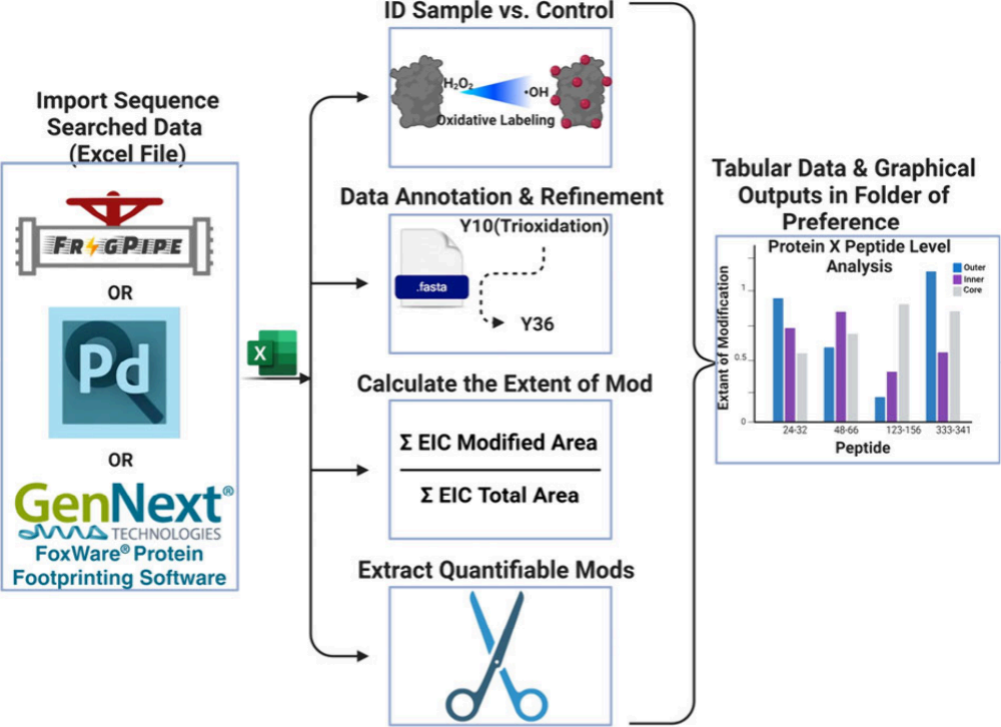
Schematic of the coADAPTr workflow. An output file from a database searching software is input into coADAPTr for quantitative analysis.

We developed coADAPTr to overcome these issues with the PP data analysis pipeline. The coADAPTr workflow where outputs from database searching software are input for quantitation is shown in Figure 1. The first challenge was to emulate the formulas from the PP pipeline in R. The PP pipeline calculates the geometric mean for the extent of FPOP modification. This requires a large number of formulas, including the transition of the data to the natural logarithmic scale and then back to the original scale (Tables S2–S3). Since geometric mean is used when the population counts are extremely variable and we do not usually see this with FPOP data, we decided to use the arithmetic mean rather than the geometric mean for coADAPTr, which reduced the number of formulas needed for the calculation of EOM. Other improvements made in coADAPTr were the addition of several filters. A filter was needed specifically for the Proteome Discoverer multilevel search workflow to remove any duplicate modifications that can come from different nodes. This particularly effects the identification of unmodified peptides as modification types are specific to a certain node. Duplicate identification of the unmodified peak will lead to a lower than actual value in the calculation of the EOM (eq 1). Though this filter was initially added for the multinode PD search, it would be useful for other search engines that may provide duplicate search results. There were other filters added to remove instances where there is no data for the sample unoxidized area, and an instruction that EOM can only be calculated when the oxidized area is observed in more than two replicate samples. Finally, because coADAPTr reads PSM level results from both PD and FragPipe, all processing of integrated peaks, including summing over multiple charge states of a peptide, can be done identically despite the different search engines. Taken together, these changes improve the reliability of the calculated EOM.

### Input required for coADAPTr

The minimal data required for input into coADAPTr postdatabase searching includes five columns of data: Master Protein Accessions, Modifications, Sequence or Peptide, Precursor Abundance, and Spectrum File (Figure S1). The Modifications column should indicate both the modification type and position. For tandem mass tag (TMT) labeling, the mass tag is used for precursor abundance. The spectrum file column indicates if an entry was from a sample file or a control file (Figure S2). The formulas used in coADAPTr specifically call on the names of these columns to execute the arithmetic. The column names are taken from the format that is used by PD and exported as an excel file, but data from other search software can also be input and processed with some changes. coADAPTr efficiently supports both label-free quantitation (LFQ) and TMT data analysis through two distinct workflows. To date, these workflows accommodate sequence-searched data from PD, FragPipe, and FoxWare Software. For initial studies with FoxWare Software, we manually manipulated the sequence, abundance, file ID, and modification columns from an exported Excel spreadsheet to meet the minimum column requirements for coADAPTr. However, to enhance user interaction and increase throughput, we have refined the data preprocessing steps within the package. Now, users can actively select and rename necessary columns—a process facilitated by on-screen prompts—prior to calculating the extent of modification. To make FragPipe files compatible with coADAPTr, a new column was added to the FragPipe output psm.tsv files by an in-house Python 3.9 script, which uses pandas, tkinter, and PyQt5 libraries, that indicated only FPOP modifications. Though we have only used three different database searching softwares to date, we expect coADAPTr to be compatible with other software as well.

### Using coADAPTr for In Vitro Data Analysis

**Figure 2:**
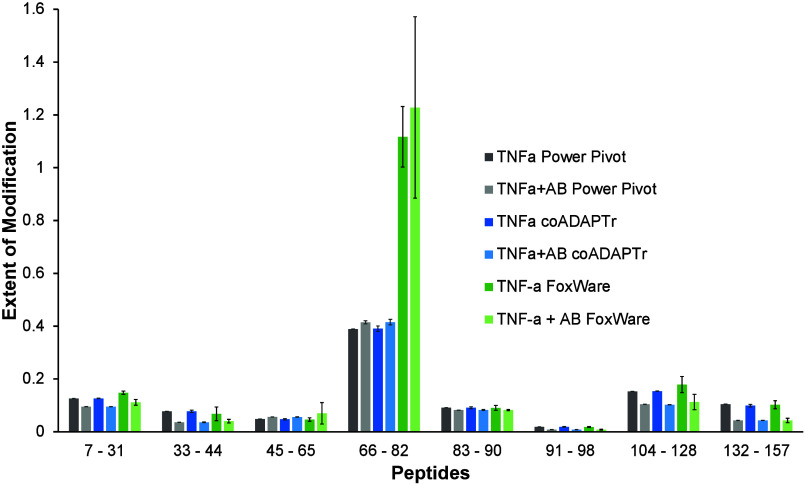
Comparison of coADAPTr, FoxWare Software, and PowerPivot. A comparison of the quantitation of TNFa alone (darker shade) and bound to Adalimumab (AB) (lighter shade) provided by GenNext Technologies. Quantitation was carried out by PowerPivot (gray bars), coADAPTr (blue bars), and FoxWare Software (green bars).

To evaluate the performance of coADAPTr, we first tested the functions against previously published in vitro FPOP data from GenNext Technologies where they successfully mapped the epitope of TNFα to Adalimumab using their flash oxidation (Fox) system.^22^ After MS analysis, they used their FoxWare Software, which is intended to be used in tandem with Fox experiments and is not openly accessible to calculate the EOM. The data from GenNext was imported into R and the coADAPTr functions were applied to the data. To generate a crosswise comparison of data outputs, the data was also subjected to analysis by PP to determine if there were differences in analysis outcomes between the three strategies (Figure 2). The analysis in PP took ∼2.5 h; including time to generate the bar graphs. Meanwhile, the coADAPTr analysis took about 5 min, a 25x higher efficiency. The FoxWare software took ∼1 h to analyze all the replicate files in this study making coADAPTr 12x more efficient.

The calculation comparison between coADAPTr and PP showed no statistically significant differences between calculated values, which validates that the arithmetic is identical, despite using the arithmetic mean for coADAPTr rather than the geometric mean. This indicates that the geometric mean is not needed for analyzing FPOP data. The calculation comparison between coADAPTr and GenNext’s data did convey some minor differences that were found to not be statistically significant. Specifically, coADAPTr reported lower EOM values for peptides 7–31, 33–44, 66–82, and 104–128 with the largest and statistically significant difference being observed for peptide 66–82. This is due to the difference in EOM calculations by FoxWare Software which calculates the average peptide oxidation (APO) events per peptide (eq 2). In peptide 66–82, there were three residues modified, and the numerator of eq 2 was multiplied by this number to achieve the APO. In contrast, coADAPTr quantifies the EOM without considering the number of modified residues per peptide in peptide-level quantitation. Instead, residue-level analysis is used by coADAPTr to account for this. Currently, FoxWare only reports on peptide-level analysis. Overall, coADAPTR’s results aligned well with the TNFα-Adalimumab epitope mapping data provided by GenNext and was within 0.01% variance, excluding peptide 66–82.

### Using coADAPTr for Proteome-Wide Data Analysis

**Figure 3:**
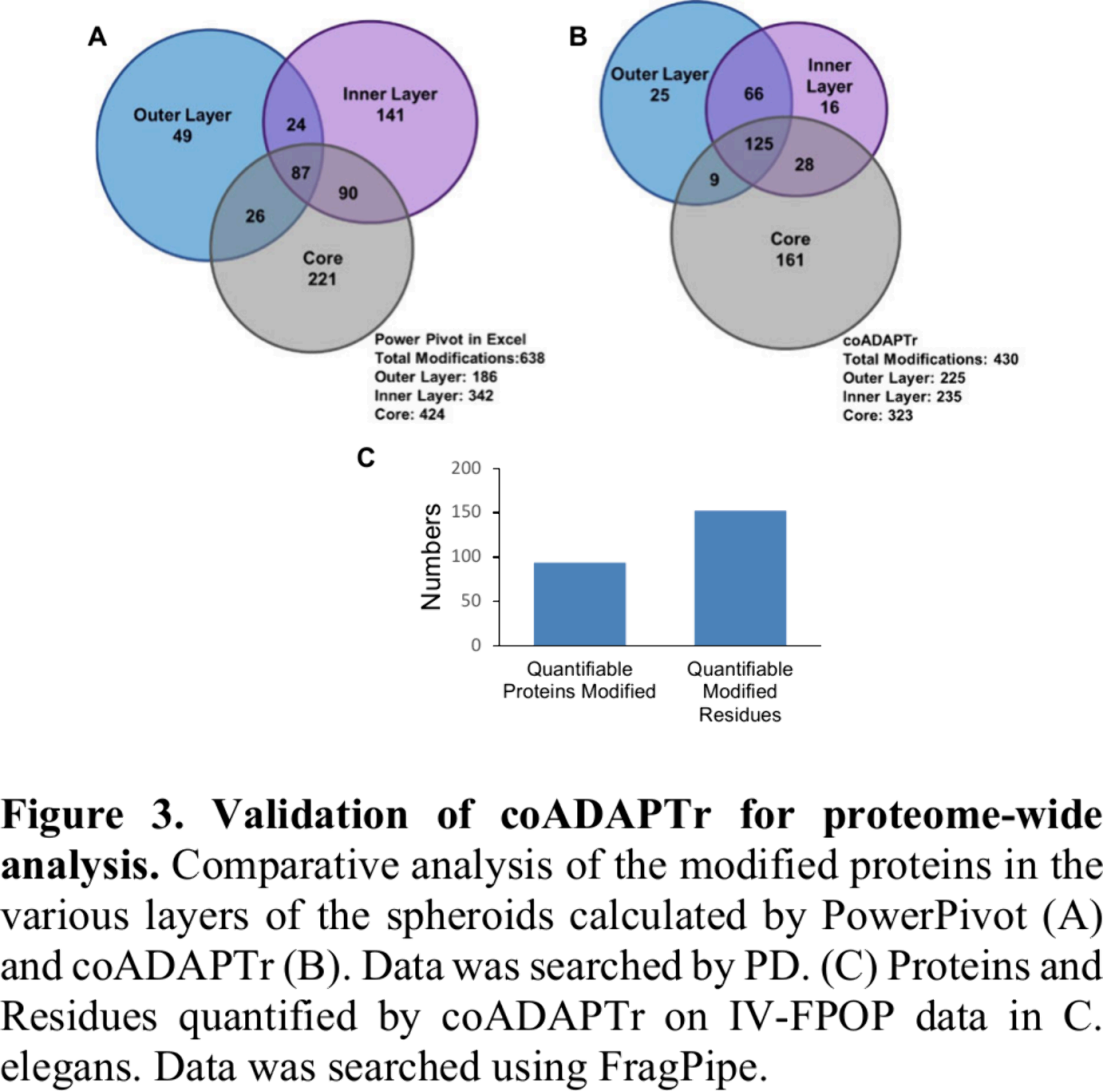
Validation of coADAPTr for proteome-wide analysis. Comparative analysis of the modified proteins in the various layers of the spheroids calculated by PowerPivot (A) and coADAPTr (B). Data was searched by PD. (C) Proteins and Residues quantified by coADAPTr on IV-FPOP data in *C. elegans*. Data was searched using FragPipe.

To evaluate the performance of coADAPTr for proteome-wide data analysis we used the results of our previously published Spheroid-FPOP data.^21^ In Spheroid-FPOP, a three-dimensional mass of cells that mimic tumors were modified. Over 600 oxidatively modified proteins were observed with over 180 modifications in each of the three layers analyzed after serial trypsinization.^3^ The excel files corresponding to the outer, inner, and core layers exported from PD were input into coADAPTr and subsequently analyzed. A Venn diagram of the modification distribution per layer was generated to compare that to the data analyzed in PP (Figure 3). The first observation noted was the decrease in the total number of proteins modified. PP calculated 638 while coADAPTr had 430. Furthermore, the number of modifications per layer was over 200 for each, while the number of proteins modified in common between the layers was higher in coADAPTr. There are a few reasons the data differ so significantly. One being that the duplicates generated by the multilevel sequence searching algorithm in PD are removed in coADAPTr before the EOM is calculated. This would result in some peptides having a lesser EOM and others may have insufficient data to quantify the extent of modification. Additionally, the filters that are implemented to ensure the reported data represent a true modification naturally reduce the number of peptides and proteins that would be counted as modified. In looking at specific proteins that were detected to be modified by both analysis methods, PP seemed to overestimate the EOM since either the peptide had duplicate entries from PD or EOM was calculated despite all the criteria not being met to quantify the EOM. Regarding analysis efficiency, the Spheroid-FPOP data took over 2 months to analyze since over 50 proteins were quantified at the peptide- and residue-levels, and graphs were generated for relevant proteins. coADAPTr generated all the peptide- and residue- level data and their corresponding graphs in ∼30 min, demonstrating robust improvements in processing timelines.

A similar time frame for quantitative analysis was also observed for an IV-FPOP data set. We used coADAPTr to analyze a *C. elegans* data set that had previously been searched using FragPipe.^16^ As mentioned earlier, a script was added in order to make the FragPipe output files compatible with the requirements for coADAPTr input. coADAPTr was able to quantify 153 modified residues across 94 proteins in ∼30 min (Figure 3C).

### Data Visualization

**Figure 4:**
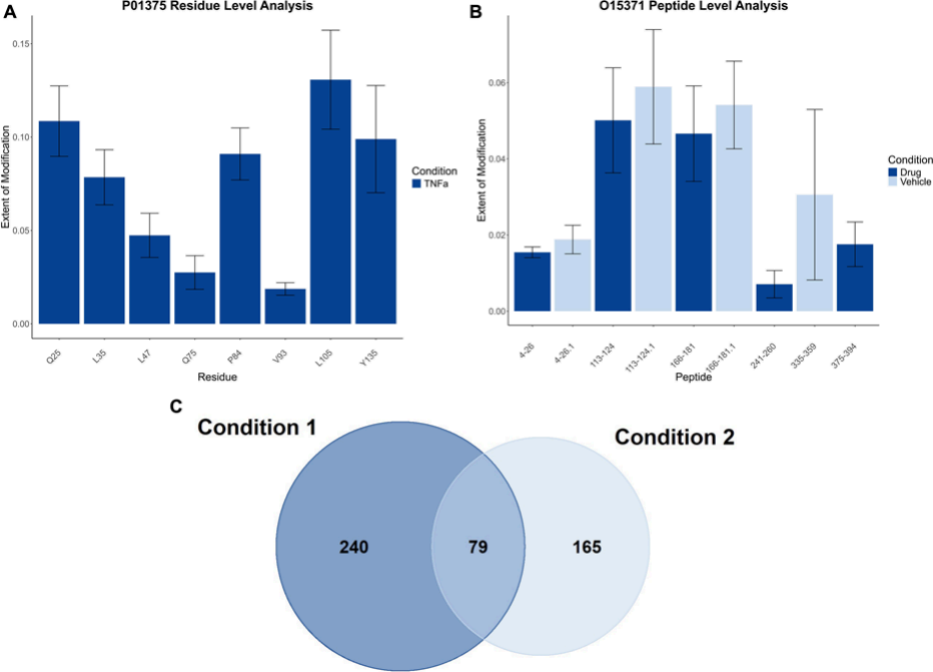
Data visualization in coADAPTr. Demonstration of the visualization capabilities of coADAPTr which includes (A) bar graphs, (B) grouped bar graphs, and (C) Venn diagrams.

To facilitate data interpretation, it is important to have a rapid visualization of the quantification results. HRPF data is most reported via a group bar graph comparing multiple states as in Figure 2. proteome-wide studies have also used Venn diagrams for data reporting. We wanted to provide users options for data visualization in coADAPTr, so it has three different graphical outputs: bar graphs for peptide- and residue-level analysis, grouped bar graphs for differential experimental conditions, and Venn diagrams for comparing oxidation between conditions (Figure 4). These graphical outputs allow for the interpretation of the experimental results to be achieved faster. Other visualization formats will be added in the future. Volcano plots, in particular, would be extremely useful for proteome-wide studies.

## Conclusion

The development of coADAPTr represents a significant advancement in the field of covalent labeling experiments such as HRPF-MS. Through the utilization of R programming, we have created a versatile and efficient platform capable of handling both in vitro and proteome-wide data analysis with ease. By emulating existing Excel PP functions and streamlining the analysis process, coADAPTr provides researchers with a powerful tool for interpreting complex structural biology data. Our validation studies demonstrate the accuracy and efficiency of coADAPTr compared to traditional methods, highlighting its potential to revolutionize data analysis in the field of structural proteomics. With its open-source availability, user-friendly interface, and comprehensive analytical capabilities, coADAPTr is poised to become an invaluable resource for researchers investigating macromolecular interactions and structural dynamics. Currently, coADAPTr is compatible with three different database searching softwares that have been used for analyzing HRPF data. Future work will focus on demonstrating the compatibility of coADAPTr with other database searching software. In addition, the software may also be adjustable to analyze different covalent labeling methods. Many covalent labeling methods modify proteins more specifically than HRPF. Adapting coADAPTr for other types of labeling should not be as challenging as the nonspecific HRPF data.

## Supporting Information Available

Table S1: Potential HRPF modifications. Table S2: Peptide-level data analysis formulas for PowerPivot. Table S3: Residue-level data analysis formulas for PowerPivot. Figure S1: Proteome Discoverer label-free quantitation input into coADAPTr. Figure S2: Protein footprinting with TMT labeling FragPipe input into coADAPTr (PDF)

## References

[ref1] Kaur U.; Johnson D. T.; Chea E. E.; Deredge D. J.; Espino J. A.; Jones L. M. Evolution of Structural Biology through the Lens of Mass Spectrometry. Anal. Chem. 2019, 91 (1), 142–155. 10.1021/acs.analchem.8b05014. 30457831 PMC6472977

[ref2] Limpikirati P.; Liu T.; Vachet R. W. Covalent labeling-mass spectrometry with non-specific reagents for studying protein structure and interactions. Methods 2018, 144, 79–93. 10.1016/j.ymeth.2018.04.002. 29630925 PMC6051898

[ref3] Chen J.; Cui W.; Giblin D.; Gross M. L. New protein footprinting: fast photochemical iodination combined with top-down and bottom-up mass spectrometry. J. Am. Soc. Mass Spectrom. 2012, 23 (8), 1306–18. 10.1007/s13361-012-0403-1. 22669760 PMC3630512

[ref4] Gau B.; Garai K.; Frieden C.; Gross M. L. Mass spectrometry-based protein footprinting characterizes the structures of oligomeric apolipoprotein E2, E3, and E4. Biochemistry 2011, 50 (38), 8117–26. 10.1021/bi200911c. 21848287 PMC3177987

[ref5] Jones L. M.; Sperry J. B.; Carroll J. A.; Gross M. L. Fast Photochemical Oxidation of Proteins for Epitope Mapping. Anal. Chem. 2011, 83 (20), 7657–7661. 10.1021/ac2007366. 21894996 PMC3193551

[ref6] Li J.; Wei H.; Krystek S. R. Jr.; Bond D.; Brender T. M.; Cohen D.; Feiner J.; Hamacher N.; Harshman J.; Huang R. Y.; Julien S. H.; Lin Z.; Moore K.; Mueller L.; Noriega C.; Sejwal P.; Sheppard P.; Stevens B.; Chen G.; Tymiak A. A.; Gross M. L.; Schneeweis L. A. Mapping the Energetic Epitope of an Antibody/Interleukin-23 Interaction with Hydrogen/Deuterium Exchange, Fast Photochemical Oxidation of Proteins Mass Spectrometry, and Alanine Shave Mutagenesis. Anal. Chem. 2017, 89 (4), 2250–2258. 10.1021/acs.analchem.6b03058. 28193005 PMC5347259

[ref7] Poor T. A.; Jones L. M.; Sood A.; Leser G. P.; Plasencia M. D.; Rempel D. L.; Jardetzky T. S.; Woods R. J.; Gross M. L.; Lamb R. A. Probing the paramyxovirus fusion (F) protein-refolding event from pre- to postfusion by oxidative footprinting. Proc. Natl. Acad. Sci. U. S. A. 2014, 140898311 10.1073/pnas.140898311. PMC4078851 24927585

[ref8] Sharp J. S.; Becker J. M.; Hettich R. L. Analysis of protein solvent accessible surfaces by photochemical oxidation and mass spectrometry. Anal. Chem. 2004, 76 (3), 672–83. 10.1021/ac0302004. 14750862

[ref9] Maleknia S. D.; Ralston C. Y.; Brenowitz M. D.; Downard K. M.; Chance M. R. Determination of macromolecular folding and structure by synchrotron x-ray radiolysis techniques. Anal. Biochem. 2001, 289 (2), 103–15. 10.1006/abio.2000.4910. 11161303

[ref10] Kiselar J. G.; Datt M.; Chance M. R.; Weiss M. A. Structural analysis of proinsulin hexamer assembly by hydroxyl radical footprinting and computational modeling. J. Biol. Chem. 2011, 286 (51), 43710–43716. 10.1074/jbc.M111.297853. 22033917 PMC3243561

[ref11] Johnson D. T.; Punshon-Smith B.; Espino J. A.; Gershenson A.; Jones L. M. Implementing In-Cell Fast Photochemical Oxidation of Proteins in a Platform Incubator with a Movable XY Stage. Anal. Chem. 2020, 92 (2), 1691–1696. 10.1021/acs.analchem.9b04933. 31860269 PMC7944481

[ref12] Espino J. A.; Jones L. M. Illuminating Biological Interactions with in Vivo Protein Footprinting. Anal. Chem. 2019, 91 (10), 6577–6584. 10.1021/acs.analchem.9b00244. 31025855 PMC6533598

[ref13] Rinas A.; Espino J. A.; Jones L. M. An efficient quantitation strategy for hydroxyl radical-mediated protein footprinting using Proteome Discoverer. Anal Bioanal Chem. 2016, 408 (11), 3021–31. 10.1007/s00216-016-9369-3. 26873216

[ref14] Sharp J. S.; Chea E. E.; Misra S. K.; Orlando R.; Popov M.; Egan R. W.; Holman D.; Weinberger S. R. Flash Oxidation (FOX) System: A Novel Laser-Free Fast Photochemical Oxidation Protein Footprinting Platform. J. Am. Soc. Mass Spectrom. 2021, 0c00471 10.1021/jasms.0c00471. PMC8812269 33872496

[ref15] Kaur P.; Kiselar J. G.; Chance M. R. Integrated algorithms for high-throughput examination of covalently labeled biomolecules by structural mass spectrometry. Anal. Chem. 2009, 81 (19), 8141–9. 10.1021/ac9013644. 19788317 PMC2764328

[ref16] Rojas Ramirez C.; Espino J. A.; Jones L. M.; Polasky D. A.; Nesvizhskii A. I. Efficient Analysis of Proteome-Wide FPOP Data by FragPipe. Anal. Chem. 2023, 95 (44), 16131–16137. 10.1021/acs.analchem.3c02388. 37878603

[ref17] Cornwell O.; Radford S. E.; Ashcroft A. E.; Ault J. R. Comparing Hydrogen Deuterium Exchange and Fast Photochemical Oxidation of Proteins: a Structural Characterisation of Wild-Type and DeltaN6 beta2-Microglobulin. J. Am. Soc. Mass Spectrom. 2018, 29 (12), 2413–2426. 10.1007/s13361-018-2067-y. 30267362 PMC6276068

[ref18] Zakopcanik M.; Kavan D.; Novak P.; Loginov D. S. Quantifying the Impact of the Peptide Identification Framework on the Results of Fast Photochemical Oxidation of Protein Analysis. J. Proteome Res. 2024, 23 (2), 609–617. 10.1021/acs.jproteome.3c00390. 38158558 PMC10845142

[ref19] Liu X. R.; Rempel D. L.; Gross M. L. Protein higher-order-structure determination by fast photochemical oxidation of proteins and mass spectrometry analysis. Nat. Protoc 2020, 15 (12), 3942–3970. 10.1038/s41596-020-0396-3. 33169002 PMC10476649

[ref20] Ziemianowicz D. S.; Sarpe V.; Schriemer D. C. Quantitative Analysis of Protein Covalent Labeling Mass Spectrometry Data in the Mass Spec Studio. Anal. Chem. 2019, 91 (13), 8492–8499. 10.1021/acs.analchem.9b01625. 31198032

[ref21] Shortt R. L.; Wang Y.; Hummon A. B.; Jones L. M. Development of Spheroid-FPOP: An In-Cell Protein Footprinting Method for 3D Tumor Spheroids. J. Am. Soc. Mass Spectrom. 2023, 34 (3), 417–425. 10.1021/jasms.2c00307. 36700916 PMC9983004

[ref22] Chea E. Laser-free flash oxidation (Fox) hydroxyl radical protein footprinting system accurately maps the paratope and epitope of TNFα bound to adalimumab. Biophys. J. 2022, 121 (3), 298A.

