# Supplementary material for: Covalent Labeling Automated Data Analysis Platform for High Throughput in R (coADAPTr): A Proteome-Wide Data Analysis Platform for Covalent Labeling Experiments
Source: J Am Soc Mass Spectrom. 2024 Oct 2;35(12):3301–7. doi: 10.1021/jasms.4c00196 (PMC11622367; doi:10.1021/jasms.4c00196)
Supplement: Supplementary file 1 — js4c00196_si_001.pdf [file js4c00196_si_001.pdf]

Supporting Information

Covalent Labeling Automated Data Analysis Platform for High Throughput in R (coADAPTr): A Proteome-Wide Data Analysis Platform for Covalent Labeling Experiments

Raquel L. Shortt,[†] Lindsay K. Pino,[‡] Emily E. Chea,[§] Carolina Rojas Ramirez,[¥] Daniel A. Polasky,[¥] Alexey I. Nesvizhskii,[¥] Lisa M. Jones[∫*]

[†]Department of Pharmaceutical Sciences, University of Maryland, Baltimore, MD 21201, United States

[‡]Talus Bio, Seattle, Washington 98122, United States

[§]GenNext Technologies, Half Moon Bay, CA 94019, United States

[¥] Department of Pathology, University of Michigan, Ann Arbor, MI, 48109, United States

[∫*]Department of Chemistry and Biochemistry, University of California San Diego, La Jolla, California, 92093, United States

*Correspondence to:
Lisa M. Jones
Department of Chemistry and Biochemistry
University of California San Diego, La Jolla, CA 92093
Phone number: 858-534-6455
Email: lijones@ucsd.ed

Table S1. Potential HRPF Modifications

Side Chain	Mass Change
Cys	+48, +32, -16
Met	+16, +32, -32
Trp	+16, +32, +48, -32
Tyr	+16, +32
Phe	+16, +32
His	+16, -23, -22, -10, +5
Leu	+16, +14
Ile	+16, +14
Val	+16, +14
Pro	+16, +14
Arg	+16, +14, -43
Lys	+16, +14
Glu	+16, +14, -30, -44
Gln	+16, +14
Asp	+16, -30, -44
Asn	+16
Ser	+16, -2
Thr	+16, -2
Ala	+16

Table S2. Peptide-Level Data Analysis Formulas for Power Pivot

Measure Name	Formula	
Area	SUM([Precursor Abundance])	Sum the precursor abundance for each sequence of amino acids
Oxidized Area	CALCULATE([Area],Data[FPOP]="Oxidized")	Calculate the area data fpop oxidized for each sequence of amino acids
Oxidation	if([Oxidized Area]>0,DIVIDE([Oxidized Area],[Area],0)+1,0)	Calculate the ratio indicating the extent of oxidation by Oxidized Area by the Total Area. If Oxidized Area is greater than 0, it adds 1 to the ratio to avoid dividing by zero. each sequence.
Oxidtn	[Oxidized Area]/[Area]	Essentially the same as Oxidation but without the conditional addition of 1. It is Oxidized Area divided by Total Area for each sequence.
LN Oxidation	if(ISERROR(LN([Oxidation])),0,LN([Oxidation]))	Calculate the Natural logarithm of the Oxidation ratio.
LN Oxidation Sq	[LN Oxidation]^2	Square of the natural logarithm of the Oxidation ratio.
N Oxidation	COUNTX(VALUES(Data[Spectrum File]),[Oxidtn])	Count the oxidized precursors for each sequence.
R	DIVIDE(SUMX(VALUES(Data[Spectrum File]),[LN Oxidation]),[N Oxidation],0)	Calculate the the mean of the natural logarithm of Oxidation ratios, divided by the count of oxidized precursors.
SA	if(ISERROR(SUMX(VALUES(Data[Spectrum File]),[LN Oxidation Sq])/[N Oxidation]),0,SUMX(VALUES(Data[Spectrum File]),[LN Oxidation Sq])/[N Oxidation])	Calculate the squared average of the natural logarithm of Oxidation ratios, divided by the count of oxidized precursors.
SB	if(ISERROR(SUMX(VALUES(Data[Spectrum File]),[LN Oxidation])/[N Oxidation])^2,0,(SUMX(VALUES(Data[Spectrum File]),[LN Oxidation])/[N Oxidation])^2)	Calculate the square of the mean of the natural logarithm of Oxidation ratios, divided by the count of oxidized precursors.
S	[SA]-[SB]	Subtract SA from SB
GeoMean	if([R]>0,EXP([R])-1,0)	Calculate the Grometric Mean of the oxidation ratios
GSD	EXP([S]+[R])-EXP([R])	Calculate the geometric standard deviation using the values obtained from SA and SB.
**Sample GeoMean**	**CALCULATE([GeoMean],Data[SampleControl]="Sample")**	Calculate the Geometric Mean for the Control group
**Control GeoMean**	**CALCULATE([GeoMean],Data[SampleControl]="Control")**	Calculate the Geometric Mean for the Sample group
**Sample GSD**	**CALCULATE([GSD],Data[SampleControl]="Sample")**	Calculate the Geometric Standard Deciation for the Sample group
**Control GSD**	**CALCULATE([GSD],Data[SampleControl]="Control")**	Calculate the Geometric Standard Deciation for the Control group
**FPOP Oxidation**	**if([Sample GeoMean]>[Control GeoMean],[Sample GeoMean]-[Control GeoMean],0)**	Calculate the extent of modification by taking the difference between the Sample and Control Geometric Means.
**FPOP GSD**	**if([FPOP Oxidation]>0,SQRT([Sample GSD]^2+[Control GSD]^2),0)**	Calculate the modified standard deviation by taking the difference between the Sample and Control Geometric Standard Deviation.
N	COUNT([Precursor Abundance])	Count the precursor abundance for each sequence.

Table S3. Residue-Level Data Analysis Formulas for PowerPivot

Data	Measures	
A	SUM([Precursor Abundance])	Calculate the total abundance of a precursor
Area	CALCULATE([A],Data[Mod Count]=0)	This calculates the precursor abundance for each residue where the modification count is 0. (ModCount counts the number of semicolons, so if there is no semicolon there is 1 modification.
Count	COUNT([Precursor Abundance])	Count the precursor sbundance
N	CALCULATE([Count],Data[Mod Count]=0)	Calculate Count for Mod Count of 0
Sample N	CALCULATE([N],Data[SampleControl]="Sample")	Count eh numberof precursors for each residue where the modification count is 0, considering only the sample group.
N Area	COUNTX(VALUES(Data[Spectrum File]),[Area])	Count of areas (abundance) for each spectrum file.
TA	CALCULATE(SUM([Precursor Abundance]),ALLEXCEPT(Data,Data[Position],Data[Condition],Data[SampleControl],Data[Spectrum File]))	Calculate the total precursor abundance, excluding specific dimensions like position, condition, sample control, and spectrum file.
Total Area	CALCULATE([TA],Data[Mod Count]=0)	Calculate the total abundance of precursors where the modification count is 0.
**Area**		
Sum Area	SUM(Area[Area])	Sum of the areas (abundance) for all observations.
Sum Total Area	SUM(Area[Total Area])	Sum of the total areas (abundance) for all observations.
Pct Area	[Sum Area]/[Sum Total Area]	Calculate the percentage of area compared to the total area.
**Percent Area**	**if([Sum Area]>0,DIVIDE([Sum Area],[Sum Total Area],0)+1,0)**	Calculation of the percentage of area. It adds 1 if the sum area is greater than 0 to avoid dividing by zero.
LN Percent Area	if(ISERROR(LN([Percent Area])),0,LN([Percent Area]))	Natural logarithm of the percentage area, handling errors if the percentage area is zero.
N Pct	COUNTX(VALUES(Area[Search ID]),[Pct Area])	Count of percentage areas for each search ID.
S	STDEVX.P(VALUES(Area[Search ID]),[LN Percent Area])	Standard deviation of the natural logarithms of percentage areas for each search ID.
R	AVERAGEX(VALUES(Data[Search ID]),[LN Percent Area])	Mean of the natural logarithms of percentage areas for each search ID.
Sum Sample N	SUM(Area[Sample N])	Sum of Sample N values.
Sum N	SUM(Area[N])	Sum of N values.
GSD	EXP([S]+[R])-EXP([R])	Calculation of the geometric standard deviation using S and R values.
GeoMean	if([R]>0,EXP([R])-1,0)	Calculation of the geometric mean, handling cases where R is greater than 0.
Sample GSD	CALCULATE([GSD],Area[SampleControl]="Sample")	Geometric standard deviation calculated for the Sample group.
Control GSD	CALCULATE([GSD],Area[SampleControl]="Control")	Geometric standard deviation calculated for the Control group.
Sample GeoMean	CALCULATE([GeoMean],Area[SampleControl]="Sample")	Geometric mean calculated for the Sample group.
Control GeoMean	CALCULATE([GeoMean],Area[SampleControl]="Control")	Geometric mean calculated for the Control group.
FPOP Oxidation	if([Sample GeoMean]>[Control GeoMean],[Sample GeoMean]-[Control GeoMean],(0))	Calculates the difference between sample and control geometric means, with a condition to handle cases where the sample geometric mean is greater than the control geometric mean.
FPOP GSD	if([FPOP Oxidation]>0,SQRT([Sample GSD]^2+[Control GSD]^2),0)	Calculation of the geometric standard deviation for FPOP oxidation, handling cases where FPOP oxidation is greater than 0.

A

Checked	Confidenc	Identifying N	PSM Ambiguity	Sequence	Modifications	# Protein (	# Proteins	Master Pr	Protein Ac	Protein De	# Missed C	Charge	DeltaScore	DeltaCn
FALSE	High	Sequest HT (A	Unambiguous	YLIYSQVLFKGQGCPSTHVLLTHTIS	Y4(Oxidation); V7(FPOP Decarb	1	1	P01375	P01375	Tumor nec	0	3	79.0128	0.029
FALSE	High	Sequest HT (A	Unambiguous	YLIYSQVLFKGQGCPSTHVLLTHTIS	Y4(Oxidation); V7(FPOP Decarb	1	1	P01375	P01375	Tumor nec	0	3	87.0128	0
FALSE	High	Sequest HT (A	Unambiguous	YLIYSQVLFKGQGCPSTHVLLTHTIS	Y4(Oxidation); V7(FPOP Decarb	1	1	P01375	P01375	Tumor nec	0	3	95.0128	0.0156
FALSE	High	Sequest HT (A	Unambiguous	YLIYSQVLFKGQGCPSTHVLLTHTIS	Y4(Oxidation); V7(FPOP Decarb	1	1	P01375	P01375	Tumor nec	0	3	175.0128	0
FALSE	High	Sequest HT (A	Unambiguous	YLIYSQVLFKGQGCPSTHVLLTHTIS	Y4(Oxidation); V7(FPOP Decarb	1	1	P01375	P01375	Tumor nec	0	3	183.0128	0.0156
FALSE	High	Sequest HT (A	Unambiguous	YLIYSQVLFKGQGCPSTHVLLTHTIS	Y4(Oxidation); V7(FPOP Decarb	1	1	P01375	P01375	Tumor nec	0	3	191.0128	0
FALSE	High	Sequest HT (A	Unambiguous	YLIYSQVLFKGQGCPSTHVLLTHTIS	Y4(Oxidation); V7(FPOP Decarb	1	1	P01375	P01375	Tumor nec	0	3	55.0128	0.0128
FALSE	High	Sequest HT (A	Unambiguous	YLIYSQVLFKGQGCPSTHVLLTHTIS	Y4(Oxidation); V7(FPOP Decarb	1	1	P01375	P01375	Tumor nec	0	3	63.0128	0
FALSE	High	Sequest HT (A	Unambiguous	YLIYSQVLFKGQGCPSTHVLLTHTIS	Y4(Oxidation); V7(FPOP Decarb	1	1	P01375	P01375	Tumor nec	0	3	71.0128	0
FALSE	High	Sequest HT (A	Unambiguous	YLIYSQVLFKGQGCPSTHVLLTHTIS	Y4(Oxidation); V7(FPOP Decarb	1	1	P01375	P01375	Tumor nec	0	3	151.0128	0
FALSE	High	Sequest HT (A	Unambiguous	YLIYSQVLFKGQGCPSTHVLLTHTIS	Y4(Oxidation); V7(FPOP Decarb	1	1	P01375	P01375	Tumor nec	0	3	159.0128	0.0156
FALSE	High	Sequest HT (A	Unambiguous	YLIYSQVLFKGQGCPSTHVLLTHTIS	Y4(Oxidation); V7(FPOP Decarb	1	1	P01375	P01375	Tumor nec	0	3	167.0128	0
FALSE	High	Sequest HT (A	Unambiguous	YLIYSQVLFKGQGCPSTHVLLTHTIS	Y4(Oxidation)	1	1	P01375	P01375	Tumor nec	0	3	31.0128	0
FALSE	High	Sequest HT (A	Unambiguous	YLIYSQVLFKGQGCPSTHVLLTHTIS	Y4(Oxidation)	1	1	P01375	P01375	Tumor nec	0	3	39.0128	0.0088
FALSE	High	Sequest HT (A	Unambiguous	YLIYSQVLFKGQGCPSTHVLLTHTIS	Y4(Oxidation)	1	1	P01375	P01375	Tumor nec	1	3	47.0128	0
FALSE	High	Sequest HT (A	Unambiguous	YLIYSQVLFKGQGCPSTHVLLTHTIS	Y4(Oxidation)	1	1	P01375	P01375	Tumor nec	0	3	127.0128	0
FALSE	High	Sequest HT (A	Unambiguous	YLIYSQVLFKGQGCPSTHVLLTHTIS	Y4(Oxidation)	1	1	P01375	P01375	Tumor nec	0	3	135.0128	0
FALSE	High	Sequest HT (A	Unambiguous	YLIYSQVLFKGQGCPSTHVLLTHTIS	Y4(Oxidation)	1	1	P01375	P01375	Tumor nec	0	3	143.0128	0
FALSE	High	Sequest HT (A	Unambiguous	IRDVELAEEALPKKTGGPQGSRRCL	V4(Oxidation); Q19(Oxidation);	1	1	P01375	P01375	Tumor nec	0	4	72.0128	0

Rank	Search Eng	m/z [Da]	MH+ [Da]	Theo. MH-	DeltaM [p	Deltam/z [	Activation	MS Order	Isolation I	Ion Inject T	RT [min]	First Scan	Spectrum File	File ID	Quan Info	XCorr	Precursor	Apex RT [n
4	4	542.2524	1004.498	1004.499	-1.27	-0.00089	CID (Collisi	MS2	1.012654	96.278	97.9304	7357	12-06-2020-TNF-alpha-0V-NL1	1		0.67	0.00E+00	21.94
1	1	550.2524	1012.498	1012.499	-1.15	-0.00106	CID (Collisi	MS2	0	104.278	105.9304	7365	12-06-2020-TNF-alpha-0V-NL2	2		0.77	0.00E+00	21.94
4	6	558.2524	1020.498	1020.499	-1.74	-0.00161	CID (Collisi	MS2	1.883226	112.278	113.9304	7373	12-06-2020-TNF-alpha-0V-NL3	3		0.63	0.00E+00	21.94
1	1	638.2524	1100.498	1100.499	-0.55	-0.00034	CID (Collisi	MS2	4.155797	192.278	193.9304	7453	12-06-2020-TNF-alpha-715V-L1	4		0.69	0.00E+00	22.94
4	5	646.2524	1108.498	1108.499	-1.74	-0.00161	CID (Collisi	MS2	1.883226	200.278	201.9304	7461	12-06-2020-TNF-alpha-715V-L2	5		0.63	0.00E+00	22.94
1	1	654.2524	1116.498	1116.499	-1.17	-0.00073	CID (Collisi	MS2	35.5006	208.278	209.9304	7469	12-06-2020-TNF-alpha-715V-L3	6		0.72	0.00E+00	22.94
2	3	518.2524	980.4976	980.4989	-1.49	-0.00069	CID (Collisi	MS2	25.05336	72.278	73.9304	7333	12-06-2020-TNF-alpha-0V-NL1	1		0.77	0.00E+00	21.94
1	5	526.2524	988.4976	988.4989	-0.66	-0.00061	CID (Collisi	MS2	0	80.278	81.9304	7341	12-06-2020-TNF-alpha-0V-NL2	2		0.7	0.00E+00	21.94
1	5	534.2524	996.4976	996.4989	-9.37	-0.00583	EThcD (ETI	MS2	15.65752	88.278	89.9304	7349	12-06-2020-TNF-alpha-0V-NL3	3		0.55	0.00E+00	21.94
1	2	614.2524	1076.498	1076.499	-0.54	-0.0003	EThcD (ETI	MS2	60.85647	168.278	169.9304	7429	12-06-2020-TNF-alpha-715V-L1	4		0.59	0.00E+00	22.94
4	4	622.2524	1084.498	1084.499	-1.74	-0.00161	CID (Collisi	MS2	1.883226	176.278	177.9304	7437	12-06-2020-TNF-alpha-715V-L2	5		0.63	0.00E+00	22.94
1	1	630.2524	1092.498	1092.499	-1.07	-0.00067	CID (Collisi	MS2	0	184.278	185.9304	7445	12-06-2020-TNF-alpha-715V-L3	6		1.62	0.00E+00	22.94
1	1	494.2524	956.4976	956.4989	-1.97	-0.00183	CID (Collisi	MS2	0	48.278	49.9304	7309	12-06-2020-TNF-alpha-0V-NL1	1		0.94	4.36E+06	21.94
3	3	502.2524	964.4976	964.4989	-0.58	-0.00037	CID (Collisi	MS2	0	56.278	57.9304	7317	12-06-2020-TNF-alpha-0V-NL2	2		1.12	6.83E+06	21.94
1	3	510.2524	972.4976	972.4989	8.51	0.00901	CID (Collisi	MS2	4.374902	64.278	65.9304	7325	12-06-2020-TNF-alpha-0V-NL3	3		0.72	6.02E+06	21.94
1	1	590.2524	1052.498	1052.499	-1.55	-0.00143	CID (Collisi	MS2	3.148209	144.278	145.9304	7405	12-06-2020-TNF-alpha-715V-L1	4		1.24	8.52E+06	22.94
1	1	598.2524	1060.498	1060.499	-2.15	-0.0015	CID (Collisi	MS2	3.444047	152.278	153.9304	7413	12-06-2020-TNF-alpha-715V-L2	5		0.78	2.05E+07	22.94
1	1	606.2524	1068.498	1068.499	-1.55	-0.00143	CID (Collisi	MS2	0	160.278	161.9304	7421	12-06-2020-TNF-alpha-715V-L3	6		0.73	2.58E+07	22.94
1	1	535.2524	997.4976	997.4989	-0.65	-0.0004	CID (Collisi	MS2	2.131463	89.278	90.9304	7350	12-06-2020-TNF-alpha-0V-NL1	1		0.71	2.48E+06	14.94

B

	Sequence	Master Protein Accessions	Modifications	Precursor Abundance	Spectrum File	Condition	SampleControl
31	WLNRRANALLANGVELRDNQLVVPS	P01375	L2(Oxidation)	45770064	NL1	TNFa	Control
39	WLNRRANALLANGVELRDNQLVVPS	P01375	L2(Oxidation)	56089546	NL2	TNFa	Control
47	WLNRRANALLANGVELRDNQLVVPS	P01375	L2(Oxidation)	38971028	NL3	TNFa	Control
127	WLNRRANALLANGVELRDNQLVVPS	P01375	L2(Oxidation)	32539199	L1	TNFa	Sample
135	WLNRRANALLANGVELRDNQLVVPS	P01375	L2(Oxidation)	48718857	L2	TNFa	Sample
143	WLNRRANALLANGVELRDNQLVVPS	P01375	L2(Oxidation)	45584245	L3	TNFa	Sample
55	WLNRRANALLANGVELRDNQLVVPS	P01375	L2(Oxidation); R5(Oxidation)	22720819	NL1	TNFa	Control
63	WLNRRANALLANGVELRDNQLVVPS	P01375	L2(Oxidation); R5(Oxidation)	33139825	NL2	TNFa	Control
71	WLNRRANALLANGVELRDNQLVVPS	P01375	L2(Oxidation); R5(Oxidation)	14316497	NL3	TNFa	Control
151	WLNRRANALLANGVELRDNQLVVPS	P01375	L2(Oxidation); R5(Oxidation)	9970553	L1	TNFa	Sample
159	WLNRRANALLANGVELRDNQLVVPS	P01375	L2(Oxidation); R5(Oxidation)	14576595	L2	TNFa	Sample
167	WLNRRANALLANGVELRDNQLVVPS	P01375	L2(Oxidation); R5(Oxidation)	16949308	L3	TNFa	Sample
79	WLNRRANALLANGVELRDNQLVVPS	P01375	L2(Oxidation); R5(Oxidation); L16(Oxidation)	174115	NL1	TNFa	Control
87	WLNRRANALLANGVELRDNQLVVPS	P01375	L2(Oxidation); R5(Oxidation); L16(Oxidation)	0	NL2	TNFa	Control
95	WLNRRANALLANGVELRDNQLVVPS	P01375	L2(Oxidation); R5(Oxidation); L16(Oxidation)	224367	NL3	TNFa	Control
175	WLNRRANALLANGVELRDNQLVVPS	P01375	L2(Oxidation); R5(Oxidation); L16(Oxidation)	259123	L1	TNFa	Sample
183	WLNRRANALLANGVELRDNQLVVPS	P01375	L2(Oxidation); R5(Oxidation); L16(Oxidation)	145023	L2	TNFa	Sample
191	WLNRRANALLANGVELRDNQLVVPS	P01375	L2(Oxidation); R5(Oxidation); L16(Oxidation)	105134	L3	TNFa	Sample
26	LSLFSFLIVAGA	P01375	L3(Oxidation)	6160395	NL1	TNFa	Control
27	TTLFCLLHFGVIGPQREEFPR	P01375	L3(Oxidation)	2865593	NL1	TNFa	Control
34	LSLFSFLIVAGA	P01375	L3(Oxidation)	6674901	NL2	TNFa	Control
35	TTLFCLLHFGVIGPQREEFPR	P01375	L3(Oxidation)	5203717	NL2	TNFa	Control
42	LSLFSFLIVAGA	P01375	L3(Oxidation)	3821932	NL3	TNFa	Control

Figure S1. Proteome Discoverer label-free quantitation input into coADAPTr. (A) An example of the output file of protein footprinting data that was searched using Proteome Discoverer 3.0 and exported as an .xlsx file. This file can be directly input into coADAPTr to calculate the extent of modification. (B) The coADAPTr format that is required for the calculation of the extent of modification. The user will follow the R console prompts in coADAPTr and select the relevant columns in the import file from Figure S1A and rename them accordingly to yield the appropriate format for calculation of the extent of modification.

A

Spectrum	Spectrum File	Peptide	Modified Peptic	Extended I	Prev AA	Next AA	Peptide Le	Charge	Retention	Observed I	Calibrated	Observed I	Calibrated	Calculatec	Calculatec	Delta Mass	Expectatio	Hyperscor	Nextscore	PeptidePr	Number of	Number of	Protein Sta	Protein En	Intensity	FPOP Modifications
20190803_Lu	interact.pep.xml	GSAGGHGSR	n[230]GSAGGH	FGGGRGS	R	S	9	2	902.8425	1013.523	1013.523	507.7688	507.7689	1013.521	507.7676	0.0027	0.011967	15.12	8.679	0.2974	2	0	23	31	406153.8	
20190803_Lu	interact.pep.xml	MAAAGGGGGGGR	n[230]MAAAGC	RRSGAMV	K	Y	12	2	946.3644	1146.579	1146.576	574.2965	574.2954	1146.577	574.2955	-0.0002	0.000109	32.449	18.269	0.9985	2	0	35	46	1881207	
20190803_Lu	interact.pep.xml	AMQGAGTQER	n[230]AMQGAC	YDAWSIRK	K	V	10	2	1011.9321	1276.641	1276.64	639.328	639.3271	1276.64	639.3271	0	0.016714	20.555	14.801	0.907	2	0	269	278	421162.3	
20190803_Lu	interact.pep.xml	GQSNQAQAR	n[230]GQSNQA	QVKQQHC	K	R	9	2	1021.4545	1187.624	1187.622	594.8193	594.818	1187.621	594.8178	0.0004	7.169232e	26.312	16.225	0.9989	2	0	567	575	2947834	
20190803_Lu	interact.pep.xml	ASQNTSSSNSSTR	n[230]ASQNTS	KKKEAAIK	K	G	13	2	1039.8082	1554.748	1554.744	778.3811	778.3795	1554.744	778.3791	0.0007	3.82e-12	44.559	13.446	1	2	0	364	376	1479115	
20190803_Lu	interact.pep.xml	NDSMSTTR	n[230]NDSM[14	HIISEISK N	K	T	8	2	1041.9549	1155.542	1155.541	578.7782	578.7779	1155.539	578.7769	0.0019	6.758262e	15.211	5.489	0.9899	2	0	225	232	1589750	4M(15.9949)
20190803_Lu	interact.pep.xml	ASQSTQAHENSR	n[230]ASQSTQ	FDPRTKPF	R	D	12	3	1050.7851	1543.756	1543.757	515.5927	515.593	1543.754	515.592	0.0029	2.476951e	34.755	16.545	1	2	0	203	214	1062455	
20190803_Lu	interact.pep.xml	TSGQTSANAHSSR	n[230]TSGQTS	IDIGNSCR	R	S	13	3	1061.1992	1531.758	1531.756	511.5933	511.5927	1531.754	511.592	0.002	2.444755e	37.893	16.388	1	2	0	422	434	1395136	
20190803_Lu	interact.pep.xml	NASCGTR	n[230]NASCGT	GSTVRQRK	K	S	7	2	1067.9586	993.4883	993.4872	497.7514	497.7509	993.4864	497.7505	0.0007	0.016321	18.268	17.776	0.8796	2	0	36	42	2622261	
20190803_Lu	interact.pep.xml	QEMQSAGSQR	n[230]QEM[147	EVKKAISK	K	G	10	2	1071.7204	1365.652	1365.65	683.8333	683.8325	1365.651	683.8327	-0.0004	2.11164e-	18.07	7.223	0.9996	2	0	205	214	3611210	3M(15.9949)
20190803_Lu	interact.pep.xml	GGCGGPR	n[230]GGCGGI	GPCWIVAI	R	A	7	2	1079.3298	888.4452	888.4449	445.2299	445.2298	888.4438	445.2292	0.0011	0.151008	13.784	9.479	0.3426	2	0	19	25	6439078	
20190803_Lu	interact.pep.xml	GGSTSSSPSR	n[230]GGSTSS	ITSFRQRK	K	R	10	2	1089.1504	1150.58	1150.579	576.297	576.2965	1150.578	576.2963	0.0004	0.003978	25.816	17.951	0.9696	2	0	65	74	2836353	
20190803_Lu	interact.pep.xml	TGDMESQR	n[230]TGDM[14	SCSAAAVI	R	D	8	2	1104.2117	1167.543	1167.541	584.7786	584.7777	1167.539	584.7769	0.0015	0.086336	11.155	8.223	0.3178	2	0	13	20	557964.9	4M(15.9949)
20190803_Lu	interact.pep.xml	SASATSSSSSSR	n[230]SASATS	SSSGGRRI	K	K	12	2	1107.0473	1342.657	1342.654	672.3358	672.3343	1342.653	672.3336	0.0013	5.116664e	34.818	14.182	1	2	0	260	271	1333672	
20190803_Lu	interact.pep.xml	RPQDGQAR	n[230]RPQDGC	REGIIGQR	R	S	8	3	1108.0989	1155.634	1155.634	386.2186	386.2187	1155.631	386.2176	0.0033	0.007812	20.096	18.198	0.3779	2	0	51	58	2511976	
20190803_Lu	interact.pep.xml	HGDGYRHPESSSR	n[230]HGDGYF	PRHGDGC	R	H	13	3	1111.2932	1712.823	1712.821	571.9481	571.9476	1712.818	571.9466	0.0029	0.024804	22.038	18.124	0.2289	2	1	840	852	1383535	
20190803_Lu	interact.pep.xml	TSAGSSSGTNSSGQR	n[230]TSAGSS	QSGHSSQ	R	H	15	2	1115.3914	1611.77	1611.768	806.8923	806.8913	1611.765	806.8898	0.0028	2.524059e	37.188	15.65	1	2	0	136	150	442673.3	
20190803_Lu	interact.pep.xml	GCGCNCSSCR	n[230]GCGCN(	WIGAIIR(	R	R	10	2	1122.2161	1445.553	1445.547	723.7836	723.781	1445.547	723.7808	0.0003	2.228435e	28.108	9.171	0.9996	2	0	14	23	601527.2	
20190803_Lu	interact.pep.xml	SGSASASGAQR	n[230]SGSASA	IKVKPPSK	K	G	11	2	1128.21	1206.618	1206.617	604.3164	604.3155	1206.616	604.315	0.0009	6.452368e	32.768	16.14	0.9999	2	0	133	143	538856.7	

Assigned Modifications	Observed I	MSFragger Localizatio	Best Score	Best Score	Purity	Is Unique	Protein	Protein ID	Entry Nam	Gene	Protein De	Mapped G	Mapped Pr	Quan Usag	VL1	VL2	VL3	VC1	VC2	DL1	DL2	NA	DL3	DC1	DC2
N-term(229.1629)					0.91	TRUE	sp| Q15056| IF4H_	Q15056	IF4H_HUM	EIF4H	Eukaryotic translation initiation fa			FALSE	805.729	1718.158	1061.679	1001.956	1646.07	1257.2	2159.473	0	1839.71	1622.824	1083.285
N-term(229.1629)					0.84	TRUE	sp| P14866| HNRF	P14866	HNRPL_HU	HNRNPL	Heterogeneous nuclear ribonucle			FALSE	13165.39	10396.11	14065.71	11098.62	13641.76	12046.63	14323.21	1171.402	14031.13	13265.76	14329.49
N-term(229.1629)					1	TRUE	sp| P20073| ANXA	P20073	ANXA7_HU	ANXA7	Annexin A7			FALSE	2676.052	1810.95	3115.791	3333.807	3046.096	3084.354	2535.07	0	3480.5	3471.009	3409.306
N-term(229.1629)					1	TRUE	sp| Q9NZI8| IF2B1	Q9NZI8	IF2B1_HUI	IGF2BP1	Insulin-like growth factor 2 mRN/			TRUE	18569.23	17339.5	34713.17	25656.94	23003.3	21930.34	19471.03	1326.475	25236.4	27457.91	38867.76
N-term(229.1629)					1	TRUE	sp| Q96KR1| ZFR_I	Q96KR1	ZFR_HUM/	ZFR	Zinc finger RNA-binding protein			FALSE	4218.516	4283.173	5626.294	4299.2	4823.104	3726.17	4061.628	0	4263.412	3601.152	3536.517
4M(15.9949), N-term(229.1629)					0.61	TRUE	sp| Q86VP6| CANI	Q86VP6	CAND1_HI	CAND1	Cullin-associated NEDD8-dissoc			FALSE	11390.76	9726.32	11391.23	13438.87	14905.38	13781.11	13316.96	1291.607	13875.16	15088.12	13716.8
N-term(229.1629)					0.93	TRUE	sp| P57737| CORC	P57737	CORO7_H	CORO7	Coronin-7			FALSE	7273.704	9929.871	11982.92	10770.91	10583.73	9188.022	7180.187	1153.531	7886.846	10352.37	7201.303
N-term(229.1629)					1	TRUE	sp| O00139| KIF2A	O00139	KIF2A_HUI	KIF2A	Kinesin-like protein KIF2A			FALSE	14551.58	26450.99	25370.51	20988.08	19723.99	22022.29	17690.55	2323.946	17867.38	23370.5	15868.02
4C(57.0214), N-term(229.1629)					1	TRUE	sp| P60468| SC61I	P60468	SC61B_HU	SEC61B	Protein transport protein Sec61 s			FALSE	11005.23	11618.93	14666.01	16750.57	13519.27	16033.94	14802.68	1212.989	14341.43	15031.53	18283.39
3M(15.9949), N-term(229.1629)					1	TRUE	sp| P51991| ROA3	P51991	ROA3_HUI	HNRNPA3	Heterogeneous nuclear ribonucle			FALSE	7867.846	8537.63	10866.74	9376.109	9621.433	10094.66	10149.4	811.1434	7504.971	9227.096	8285.475
3C(57.0214), N-term(229.1629)					0.78	TRUE	sp| P36551| HEM6	P36551	HEM6_HUI	CPOX	Oxygen-dependent coproporphy			FALSE	54486.82	60188.3	106605.2	97370.99	79481.74	78378.09	61464.84	5666.581	74391.03	99316.88	130019.1
N-term(229.1629)					0.78	TRUE	sp| Q14739| LBR_I	Q14739	LBR_HUM/	LBR	Delta(14)-sterol reductase LBR			FALSE	20791.75	19017.17	26035.8	15141.59	20496.09	22451.09	28394.37	1832.76	26680.84	17864.63	16968.27
4M(15.9949), N-term(229.1629)					0.86	TRUE	sp| Q15814| TBCC	Q15814	TBCC_HUI	TBCC	Tubulin-specific chaperone C			FALSE	9121.007	7655.036	11330.69	10011.02	11430.17	8151.013	9221.951	0	9603.907	11631.04	14217.2
N-term(229.1629)					1	TRUE	sp| Q14004| CDK1	Q14004	CDK13_HU	CDK13	Cyclin-dependent kinase 13			FALSE	0	1110.903	1282.945	675.6551	944.0145	1098.608	1827.975	0	1341.211	780.1278	1380.022
N-term(229.1629)					0.59	TRUE	sp| Q6UB35| C1TN	Q6UB35	C1TM_HUI	MTHFD1L	Monofunctional C1-tetrahydrofol			FALSE	20975.94	16382.52	29287.36	13362.33	16215.68	18828.23	20810.25	1258.236	21921.35	13718.37	16978.09
N-term(229.1629)					0.92	TRUE	sp| Q86XP3| DDX4	Q86XP3	DDX42_HU	DDX42	ATP-dependent RNA helicase DD			FALSE	9759.887	9082.681	13423.81	7570.294	8380.282	7619.129	8491.785	0	9342.461	7740.759	6571.689
N-term(229.1629)					1	TRUE	sp| Q9UHB7| AFF4	Q9UHB7	AFF4_HUM	AFF4	AF4/FMR2 family member 4			FALSE	0	0	0	0	0	660.385	0	0	0	0	0
2C(57.0214), 4C(57.0214), 6C(57.0214), 9C(57.0214), N-term(229.1629)					1	TRUE	sp| Q12849| GRSF	Q12849	GRSF1_HU	GRSF1	G-rich sequence factor 1			FALSE	4077.081	3440.808	3464.779	5440.723	5575.166	4929.792	4692.408	0	5609.854	6569.079	5600.501
N-term(229.1629)					0.72	TRUE	sp| Q8IXZ2| ZC3H3	Q8IXZ2	ZC3H3_HU	ZC3H3	Zinc finger CCCH domain-contai			FALSE	5517.994	5740.363	7641.497	3006.032	3711.533	4499.113	5780.731	0	5145.132	3530.647	4094.092

B

	Sequence	Master Protein Accessions	Modifications	Vehicle:L1	Vehicle:L2	Vehicle:L3	Vehicle:NL1	Vehicle:NL2	Drug:L1	Drug:L2	Drug:L3	Drug:NL1	Drug:
1	GSAGGHGSR	Q15056	NA	2.305213e+04	4.915696e+04	3.037493e+04	2.866624e+04	4.709452e+04	3.596882e+04	6.178311e+04	5.263462e+04	4.642944e+04	
2	MAAAGGGGGGGR	P14866	NA	1.899823e+05	1.500204e+05	2.029744e+05	1.601579e+05	1.968565e+05	1.738381e+05	2.066902e+05	2.024753e+05	1.914307e+05	
3	AMQGAGTQER	P20073	NA	3.761487e+04	2.545491e+04	4.379589e+04	4.686035e+04	4.281626e+04	4.335401e+04	3.563322e+04	4.892228e+04	4.878888e+04	
4	GQSNQAQAR	Q9NZI8	NA	2.170068e+05	2.026357e+05	4.056708e+05	2.998364e+05	2.688249e+05	2.562860e+05	2.275455e+05	2.949218e+05	3.208832e+05	
5	ASQNTSSSNSSTR	Q96KR1	NA	1.470262e+05	1.492797e+05	1.960909e+05	1.498382e+05	1.680976e+05	1.298667e+05	1.415583e+05	1.485909e+05	1.255095e+05	
6	NDSMSTTR	Q86VP6	4M(15.9949)	1.386233e+05	1.183674e+05	1.386290e+05	1.635484e+05	1.813955e+05	1.677134e+05	1.620647e+05	1.688580e+05	1.836194e+05	
7	ASQSTQAHENSR	P57737	NA	8.368162e+04	1.142400e+05	1.378596e+05	1.239159e+05	1.217624e+05	1.057052e+05	8.260573e+04	9.073562e+04	1.191007e+05	
8	TSGQTSANAHSSR	O00139	NA	9.956374e+04	1.809810e+05	1.735882e+05	1.436031e+05	1.349540e+05	1.506792e+05	1.210409e+05	1.222508e+05	1.599039e+05	
9	NASCGTR	P60468	NA	1.975898e+05	2.086083e+05	2.633161e+05	3.007426e+05	2.427273e+05	2.878761e+05	2.657699e+05	2.574884e+05	2.698787e+05	
10	QEMQSAGSQR	P51991	3M(15.9949)	3.104121e+05	3.368372e+05	4.287283e+05	3.699180e+05	3.795968e+05	3.982673e+05	4.004268e+05	2.960955e+05	3.640389e+05	
11	GGCGGPR	P36551	NA	4.168274e+05	4.604441e+05	8.155363e+05	7.448938e+05	6.080400e+05	5.995970e+05	4.702097e+05	5.690958e+05	7.597800e+05	
12	GGSTSSSPSR	Q14739	NA	2.757777e+05	2.522400e+05	3.453339e+05	2.008350e+05	2.718561e+05	2.977868e+05	3.766173e+05	3.538894e+05	2.369530e+05	
13	TGDMESQR	Q15814	4M(15.9949)	4.971232e+04	4.172232e+04	6.175580e+04	5.456315e+04	6.229796e+04	4.442556e+04	5.026250e+04	5.234428e+04	6.339280e+04	
14	SASATSSSSSSR	Q14004	NA	0.000000e+00	1.418940e+05	1.638686e+05	8.630042e+04	1.205776e+05	1.403236e+05	2.334845e+05	1.713108e+05	9.964457e+04	
15	RPQDGQAR	Q6UB35	NA	2.795576e+05	2.183387e+05	3.903284e+05	1.780870e+05	2.161150e+05	2.509340e+05	2.773494e+05	2.921576e+05	1.828321e+05	
16	HGDGYRHPESSSR	Q86XP3	NA	1.534748e+05	1.428257e+05	2.110903e+05	1.190434e+05	1.317805e+05	1.198113e+05	1.335339e+05	1.469108e+05	1.217239e+05	
17	TSAGSSSGTNSSGQR	Q9UHB7	NA	0.000000e+00	0.000000e+00	0.000000e+00	0.000000e+00	0.000000e+00	4.426733e+05	0.000000e+00	0.000000e+00	0.000000e+00	
18	GCGCNCSSCR	Q12849	NA	4.964505e+04	4.189740e+04	4.218929e+04	6.624960e+04	6.788665e+04	6.002819e+04	5.713766e+04	6.830904e+04	7.998916e+04	
19	SGSASASGAQR	Q8IXZ2	NA	6.109684e+04	6.355898e+04	8.460888e+04	3.328366e+04	4.109518e+04	4.981549e+04	6.400593e+04	5.696841e+04	3.909235e+04	
20	SSAQNVHNDR	O75449	NA	8.715223e+04	8.589631e+04	1.203179e+05	8.457895e+04	9.125740e+04	8.972335e+04	9.738082e+04	8.765764e+04	7.756357e+04	

Figure S2. Protein footprinting with TMT labeling FragPipe Input into coADAPTr. (A) An example of protein footprinting data that used TMT labeling that was searched and exported from FragPipe. Once data is converted from a .csv file to a .xlsx file it is ready to be input in coADAPTr to calculate the extent of modification. (B) The coADAPTr format that is required for the calculation of the extent of modification. The user will follow the R console prompts in coADAPTr and select the relevant columns in the import file from Figure S2A and rename them accordingly to yield the appropriate format for calculation of the extent of modification. This data will be further annotated and normalized before calculating the extent of modification.
